# Medial artery calcification in peripheral artery disease

**DOI:** 10.3389/fcvm.2023.1093355

**Published:** 2023-01-26

**Authors:** Tanner I. Kim, Raul J. Guzman

**Affiliations:** ^1^Deparment of Surgery, John A. Burns School of Medicine, University of Hawaii, Honolulu, HI, United States; ^2^The Queen’s Health Systems, Honolulu, HI, United States; ^3^Division of Vascular Surgery and Endovascular Therapy, Department of Surgery, Yale School of Medicine, New Haven, CT, United States

**Keywords:** medial artery calcification, peripheral artery (occlusive) disease, arterial calcification, arterial stiffness, chronic limb-threatening ischemia

## Abstract

Medial artery calcification (MAC) is a distinct, highly regulated process that is often identified in small and mid-sized arteries of the lower extremities. It is associated with advanced age, diabetes, and chronic kidney disease. MAC often occurs in conjunction with atherosclerotic occlusive disease in lower extremity arteries, and when seen together or in isolation, long-term limb outcomes are negatively affected. In patients with peripheral artery disease (PAD), the extent of MAC independently correlates with major amputation and mortality rates, and it predicts poor outcomes after endovascular interventions. It is associated with increased arterial stiffness and decreased pedal perfusion. New endovascular methods aimed at treating calcified lower-extremity lesions may improve our ability to treat patients with limb-threatening ischemia. Although recent developments have increased our understanding of the mechanisms contributing to MAC, further investigations are needed to understand the role of medial calcification in PAD, and to develop strategies aimed at improving patient outcomes.

## Introduction

Medial artery calcification (MAC), previously known as Monckeberg’s sclerosis, affects the peripheral arteries and is most strongly associated with diabetes and chronic kidney disease. It develops through a complex process involving the transformation of vascular smooth muscle cells (VSMC) into an osteogenic phenotype that culminates in the formation of calcium hydroxyapatite crystals between the intimal and adventitial layers (1). Recent work has demonstrated that this highly regulated process may develop independent of atherosclerosis and has features similar to that of developing bone (1–3). The process is distinct from intimal calcification that develops on the luminal side of the internal elastic lamina. The intimal form is commonly found in coronary and larger arteries throughout the body (Table 1). Based on recent studies, medial calcification is now thought to be an independent contributor to the complex pathophysiology of PAD (4–9). In this review, we discuss the relationship between MAC and PAD and its effects on long-term limb outcomes.

**Table 1:** Characteristics of intimal and medial calcification.

Intimal calcification	Medial calcification
Associated with atherosclerosis and traditional cardiovascular risk factors	Associated with diabetes, chronic kidney disease and metabolic dysfunction
Characterized by subintimal lipid deposition and macrophages	Characterized by elastin layer disruption and calcification
Can be associated with inflammatory cell infiltrate and bone formation within atherosclerotic plaque	Associated with osteogenic transformation of medial smooth muscle cells. Bone formation less common.
Appears as patchy or spotty areas of calcification on the luminal side of internal elastic lamina	Appears as smooth, continuous areas of calcification, typically seen as parallel lines or “railroad” tracks on plain x-rays.
Commonly affect coronary, carotid, and larger arteries	More commonly seen in peripheral small and medium-sized arteries
Advanced form leads to vessel stenosis or occlusion	Advanced form associated with increased arterial stiffness

### Epidemiology and risk factors

Although MAC can develop in the setting of typical atherosclerosis risk factors, it is most commonly identified in association with diabetes and chronic kidney disease (10–14). The medial calcification that arises in patients with diabetes is thought to result from multiple overlapping mechanisms including prolonged hyperglycemia, inflammation, advanced glycation end products, circulation of osteoprogenitor cells, and decreased matrix gamma-carboxyglutamic acid protein (MGP) levels (15–18). We previously reported on the risk factors for MAC in patients without renal disease (19). Increased tibial artery calcium scores were associated with age, male sex, diabetes, and tobacco use, but interestingly, not with hypertension, hyperlipidemia, or body mass index.

In patients with chronic kidney disease, medial calcification is thought to develop in part through dysregulated calcium and phosphorus metabolism as well as increased oxidative stress (1, 15). The role of hyperphosphatemia in the development of arterial calcification has been investigated (1, 20–22). Hyperphosphatemia leads to a variety of changes that affect VSMCs including calcium-phosphate deposits, promotion of an osteogenic phenotype, and apoptosis (23–26). We previously demonstrated that high serum phosphate levels are independently associated with worse amputation-free survival following intervention for chronic limb-threatening ischemia (CLTI) (22). In 941 patients with CLTI undergoing lower extremity intervention, 14% were found to have elevated phosphate levels. Patients with elevated phosphate levels had significantly higher mortality rates and decreased amputation-free survival. These results persisted after adjustment for multiple demographic and cardiovascular risk factors including age, diabetes, and chronic kidney disease.

Differing risk factor profiles have also been observed between intimal and medial artery calcification. Zwakenberg et al. pooled together two cohort studies to assess risk factors associated with lower extremity intimal and medial calcification (27). Patients with the medial form had higher rates of diabetes and elevated ankle-brachial indices, and lower rates of smoking compared to those with intimal calcification. Interestingly, the inactive form of matrix-Gla protein was associated with medial calcification, while osteonectin was associated with the intimal type. Matrix Gla protein is an inhibitor of arterial calcification, and medications that lead to decreased carboxylation of matrix Gla protein such as warfarin have also been found to promote MAC (28, 29). Alappan et al. demonstrated that warfarin use was associated with a four-fold increase in MAC progression (29). Progression of MAC was further accelerated among warfarin users with end-stage renal disease, and slowed following cessation of warfarin therapy.

Inflammatory disorders such as rheumatoid arthritis have been associated with MAC formation. This is thought to occur secondary to chronic inflammation, as elevated IL-6 levels are associated with osteogenic differentiation (30–32). The presence of MAC in patients with rheumatoid arthritis has also been shown to be a risk factor for worse all-cause mortality (33). The role of specific genotypes in the development medial calcification has recently been highlighted. St. Hilaire et al. reported on families with symptomatic arterial and joint calcification with identified mutations in the gene *NT5E* (34). This gene encodes for CD73, which is an enzyme involved in the adenosine triphosphate (ATP) metabolic pathway that has been since found to influences inorganic pyrophosphate and phosphate metabolism. Pseudoxanthoma elasticum is a rare disease that is also well associated with MAC formation (35, 36). The gene involved in this disease is *ABCC6*, which encodes for an adenosine triphosphate-binding cassette transporter primarily expressed in the liver and kidney. However, the underlying pathophysiology leading to MAC formation remains unclear (35). Generalized arterial calcification of infancy (GACI) is a rare disorder most commonly associated with mutations in the *ENPP1* gene (Ectonucleotide pyrophosphatase/phosphodiesterase family member one or less commonly the *ABCC6* gene). It is characterized by calcification and disruption of the media that can lead to stenosis or rupture (37).

These studies emphasize the multiple, overlapping risk factors that contribute to the complex pathophysiology of MAC, including inflammation, mineral metabolism, and osteogenic pathways. The development of relevant treatments will require continued investigations into its shared and distinct pathways with plaque calcification, and further efforts to understand its role in the pathogenesis of PAD.

### Medial artery calcification in peripheral arteries

**Figure 1:**
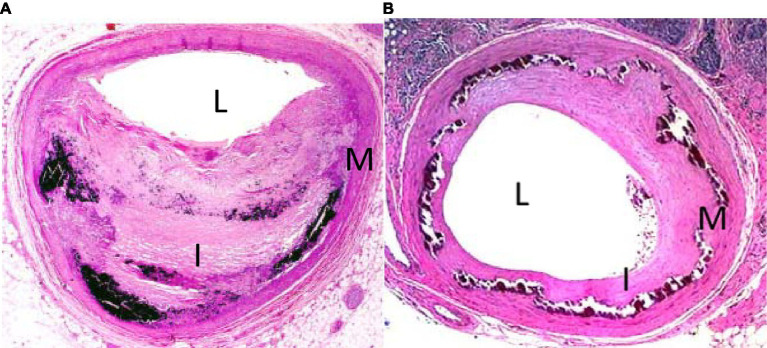
H&E stain of tibial arteries from patient with intimal **(A)** and medial **(B)** calcification. L denotes lumen, I denotes intima, M denotes media.

The histopathology of medial calcification is distinct from that of the intimal form (Figure 1). In a study performed on atherectomy specimens from symptomatic PAD patients, Krishnan et al. found that the combined presence of diabetes and chronic kidney disease was more strongly associated with MAC than individual risk factors (38). Other histological studies have demonstrated a correlation between age and MAC. Kamenskiy et al. analyzed femoropopliteal arteries from 431 donors and found that over 60% of patients over age 60 years had evidence of femoropopliteal calcification and at least half had the medial form (39). Interestingly, small calcium deposits were observed in femoropopliteal arteries of patients as young as 18 years of age.

**Figure 2:**
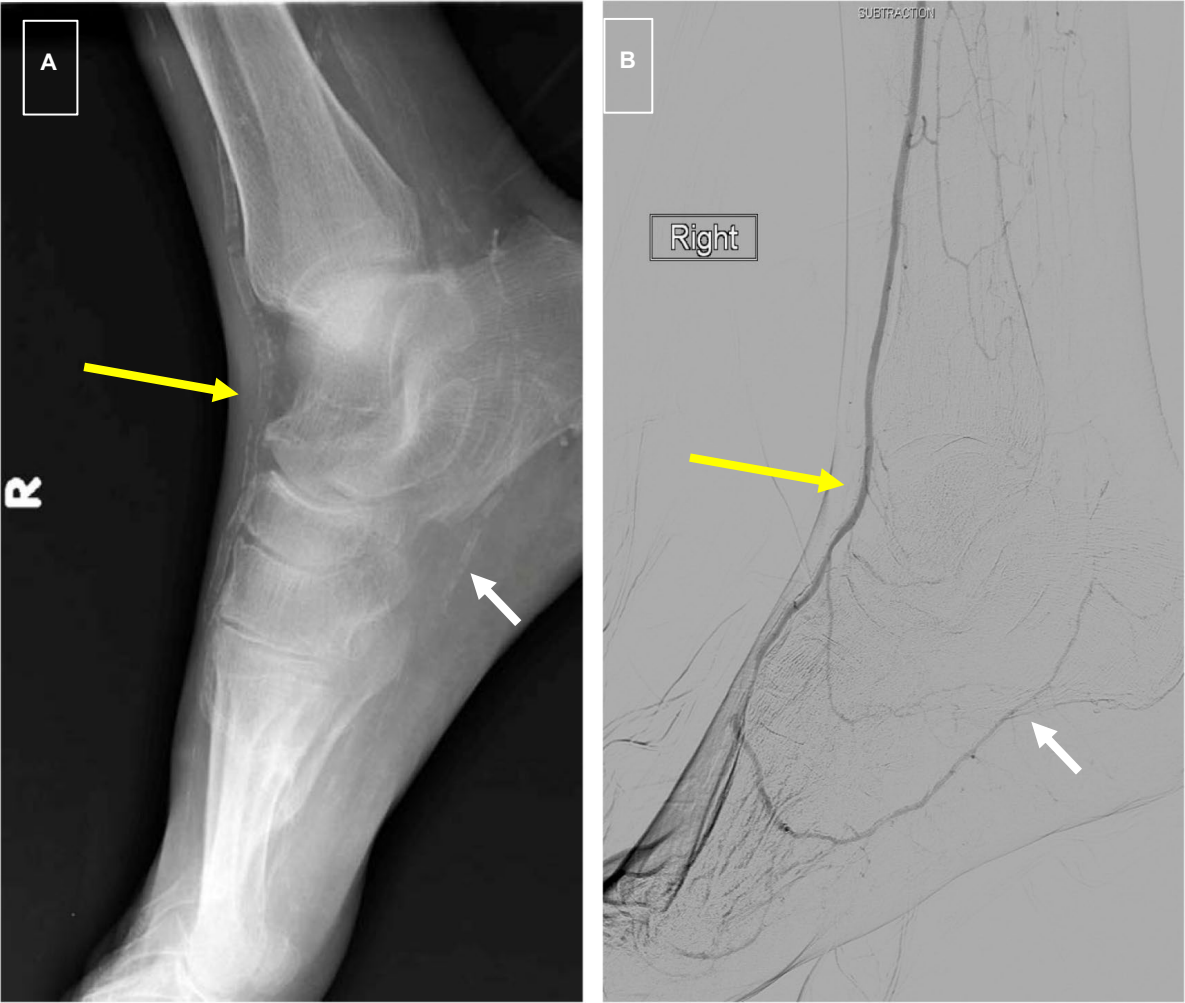
**(A)** Foot x-rays showing typical “railroad track” appearance of dorsalis pedis artery signifying medial calcification (long yellow arrow) and “patchy” appearance of intimal calcification in plantar artery (short white arrow). **(B)** Digital subtraction arteriogram from same patient showing a patent DP (long yellow arrow) as is typical of medial calcification and high-grade stenosis of plantar arteries associated with intimal calcification (short white arrow).

The association of MAC with peripheral atherosclerosis is well known. In 1988, Everhart et al. published their longitudinal findings on 4,553 Pima Indians who were followed over a period of 20 years (40). Patients with diabetes and MAC as defined by a linear appearance of calcifications on x-rays of the hands, feet, calf regions, and thighs had a 5.5-fold increased risk of major amputation, and 1.5-fold mortality rate compared to diabetes patients without it (Figure 2). More recent studies have confirmed a predictive role for MAC on limb outcomes, and several investigators have shown an independent association between it and the severity of PAD as well as the risk of major amputation (5–7). It is noteworthy that traditional tests for PAD such as the ankle-brachial index may be inaccurate in patients with calcified arteries, making it difficult to assess the true severity of ischemia using standard measures. In a study involving 116 patients with symptomatic PAD, we showed that increasing calcification scores correlated with the extent of ischemia, and that this relationship was maintained after adjustment for cardiovascular risk factors and the extent of occlusive disease (6).

Lower extremity calcification scores also correlate with the presence of foot ulceration in the setting of PAD. In a study involving 162 patients with diabetes, we showed that those with foot ulcers were more likely to have a higher tibial calcification score, and that this relationship persisted after adjustment for cardiovascular risk factors and the peripheral artery occlusion index. These findings suggest that medial calcification contributes to decreased pedal perfusion in a manner that is independent of atherosclerotic burden, and possibly *via* mechanisms related to alterations in blood vessel structure.

Tibial artery calcification is also a significant prognostic marker for PAD severity and worse limb-related outcomes. We previously investigated the relationship between tibial artery calcification, severity of limb ischemia, and risk of major amputation among 229 patients with symptomatic PAD (5). The tibial artery calcification score was found to correlate with the severity of limb ischemia at presentation, and higher levels were associated with major amputation and were better predictors of amputation than the ABI. A recent study from Liu et al. created a simple scoring system utilizing foot x-rays to score pedal artery calcification in 250 patients who underwent infra-inguinal revascularization for CLTI (41). After a mean follow up of 759 days, higher pedal calcification scores were associated with an elevated risk of major amputation. Furthermore, the calcification score was able to further stratify the risk of major amputation among high-risk patients with diabetes. These studies demonstrate a clear relationship between MAC and worse limb-related outcomes. One potential mechanistic link between medial calcification and poor limb outcomes is through its effects on arterial stiffness, which can lead to dysfunctional blood flow dynamics as discussed in the following section.

Several limitations continue to affect current clinical studies on the epidemiology and consequences of medial calcification. Importantly, it remains difficult to confidently distinguish medial from intimal calcification on x-rays and CT scans. The resolution of these commonly used methods does not provide sufficient resolution to specify which form is involved in a specific vessel segment with the most common protocols used. Additionally, peripheral arteries are commonly affected by both medial and intimal calcification in varying ratios. As such, efforts to isolate the effects of one form over the other are either not possible or severely limited. Finally, unlike measurement of calcification in the coronaries, different calcium measuring methodologies are currently in use by various investigators, and this has complicated the comparison of results. Future efforts to identify the best methods for identifying and quantifying medial artery calcification are needed.

### Medial artery calcification and arterial stiffness

MAC leads to significant structural changes within the vessel wall. The accumulation of medial calcification reduces vessel compliance and alters blood flow dynamics (42, 43). In larger arteries, the so-called Windkessel effect, an imprecise term used to describe the dampening effect of large elastic arteries on the pulse waveforms (44), causes increased pulse pressure and cardiac afterload which have been associated with left ventricular hypertrophy and heart failure (43, 45, 46). The loss of vessel wall compliance within smaller arteries has been associated with stasis and an attenuated autoregulatory response which may contribute to decreased perfusion (42). This suggests one mechanism whereby MAC can contribute to an increased risk of cardiovascular morbidity and mortality (11, 47, 48).

Arterial stiffness has also been associated with worse outcomes following lower extremity interventions for limb-threatening ischemia. We previously investigated the association between pulse pressure, as a surrogate for arterial stiffness, and endovascular interventions for CLTI (49). Among 371 patients who underwent tibial artery interventions, 185 patients had a preoperative pulse pressure of 80 mm Hg or greater signifying increased arterial stiffness. Patients with a higher pulse pressure were more likely to suffer from procedural complications that included arterial dissection, spasm, embolism, rupture, or arteriovenous fistula. Although short terms outcomes were not worse in the high pulse pressure group, five-year mortality was higher among these patients. Similarly, a pulse pressure of 80 mm Hg or greater was also found to be independently associated with worse amputation-free survival at 1 year after lower extremity bypass (50). Interestingly, histologic studies of the superficial femoral artery have shown that patients with MAC have a thinner elastic lamina with discontinuous elastic fibers potentially explaining the higher risk of procedural complications (39). Calcification is also associated with increased stiffness in both the longitudinal and circumferential directions (39, 51).

Another potential explanation for the worse outcomes seen in patients with MAC is its association with an impaired ability to recover from ischemic events. We have investigated the relationship between MAC and limb ischemia in rodent models of disease (52). Animals with MAC induced by vitamin D overload exhibited an impaired ability to recover from ischemia caused by femoral artery ligation. Animals with calcified arteries demonstrated more severe ischemia and delayed functional recovery compared with controls. Interestingly, capillary density and muscle morphology were not different between the two groups, suggesting that differences in angiogenic responses were not the primary mechanistic explanation. However, arterial stiffness was increased in animals with calcified arteries and was associated with medial elastin degradation, suggesting that MAC may lead to worse outcomes in part due to its effects on arterial compliance. Other studies have also demonstrated that decreased arterial compliance is associated with increased peripheral vascular resistance and a decreased vasodilatory response, which may contribute to decreased perfusion (53, 54). However, it is likely that other factors also contribute to the complex mechanisms behind MAC and decreased perfusion, warranting further investigation.

### Interventions for peripheral artery disease with medial artery calcification

While intimal calcification within dense atherosclerotic plaques has been known to affect technical aspects of endovascular interventions, emerging data also suggests a negative impact of MAC. Kang et al. investigated the relationship between calcium burden and tibial endovascular interventions among patients with CLTI (55). Patients with extensive tibial artery calcification had the lowest rates of technical success, and at 2 years had significantly higher rates of unplanned major amputation. Similarly, Skolnik et al. found that an increased burden of MAC in the foot and ankle by x-ray was predictive of an increased risk of major adverse limb events following tibial artery angioplasty (56). One accepted explanation for the worse outcomes is that severely calcified arteries have higher rates of elastic recoil following balloon angioplasty and limit stent expansion leading to poor patency rates (57–59). However, data in this regard are limited by the current inability to clearly distinguish medial from intimal calcification on plain X-rays and CT scans as well as the high degree of overlap between the two forms in many anatomic regions.

An additional concern is that the presence of MAC can promote the development of neointimal hyperplasia following angioplasty leading to increased levels of in-stent restenosis. Using a rodent model, we previously showed that neointimal hyperplasia was significantly increased in calcified arteries (60). Interestingly, calcified arteries also had higher levels of the bone morphogenetic protein-2 (BMP-2) and increased BMP-induced smooth muscle cell proliferation compared with non-calcified arteries. Increased BMP-2 expression and BMP-responsiveness of SMC from calcified arteries suggests that controlling the restenosis response may require different therapeutic targets in calcified arteries.

### The state of current therapies and future directions

Mechanical methods to address calcified arteries in patients with PAD are under development. Intravascular lithotripsy has recently been introduced as a novel endovascular technique to address severe calcification. This method utilizes a specialized angioplasty balloon inflated to a low atmospheric pressure with emitters that generate pulsatile mechanical energy to fracture surrounding arterial calcium while minimizing trauma to the surrounding vessel (61). Initial single arm studies of patients with heavily calcified lesions in the femoral and tibial arteries have demonstrated high rates of technical success with low complication rates and bailout stenting (61–63). Randomized studies are ongoing, with encouraging mid-term results at 1 year (64). While most attention has been focused on treatment of atherosclerotic intimal disease, studies by Brodmann et al. have shown good early results when used in tibial lesions where calcification is more likely to be medial (64).

Despite advances in our understanding of its central mechanisms, no therapies have proven effective in slowing or reversing peripheral artery calcification. Many of the underlying causes of MAC are still unknown, and it is unclear whether the expression of calcification-related genes is a driver of the adverse pathologic responses or the result of it (42). At this time, many studies involving various medications including Vitamin K, sodium thiosulfate, magnesium, calcimimetics, and bisphosphonates among others are ongoing (65–69). Investigating the genetics associated with MAC will also improve our understanding of the underlying pathophysiology and may lead to new therapeutics. MAC formation is well-described in several rare monogenetic disorders (31, 35, 36, 70). Genome-wide association studies (GWAS) may also provide further insight in the future. Several studies have already used GWAS to identify genetic associations with PAD (71, 72). Zekavat et al. performed GWAS using the United Kingdom Biobank to identify whether predisposition to increased arterial stiffness was associated with hypertension and coronary artery disease (73). Rare diseases and new technologies provide an opportunity to further our understanding of the genetic and pathophysiologic mechanisms underlying MAC and will hopefully lead to new treatment options in the future.

## Conclusion

MAC is a common yet complex process that is often seen in patients with PAD. Increased medial calcification levels correlate with worse limb and cardiovascular outcomes in a manner that appears to be independent of atherosclerosis. It is associated with increased arterial stiffness and decreased pedal perfusion and it limits the durability of endovascular interventions. New therapies are currently under development for the treatment of calcified vessels. Further research is needed to better understand the role of MAC in PAD, and to identify newer treatments aimed at preventing it and improving outcomes for our vascular patients.

## Author contributions

TK and RG contributed to the writing of the manuscript. All authors contributed to the article and approved the submitted version.

## Funding

This work was supported by R01 HL138357 and R01 HL157111.

## Conflict of interest

The authors declare that the research was conducted in the absence of any commercial or financial relationships that could be construed as a potential conflict of interest.

## Publisher’s note

All claims expressed in this article are solely those of the authors and do not necessarily represent those of their affiliated organizations, or those of the publisher, the editors and the reviewers. Any product that may be evaluated in this article, or claim that may be made by its manufacturer, is not guaranteed or endorsed by the publisher.
